# Intrapleural Gentian Violet Irrigation for Refractory Methicillin‐Resistant *Staphylococcus aureus* Empyema: A Case Series

**DOI:** 10.1002/rcr2.70562

**Published:** 2026-03-26

**Authors:** Yasoo Sugiura, Hiroyuki Fujimoto, Yoshihiko Morikawa, Kosuke Sugino, Shun Yorimori, Arihito Tanaka, Toshinori Hashizume, Morio Nakamura

**Affiliations:** ^1^ Department of General Thoracic Surgery National Hospital Organization, Kanagawa Hospital Hadano City Kanagawa Japan; ^2^ Research Promotion Center, Tokyo Metropolitan Hospital Organization Fuchu‐shi Tokyo Japan; ^3^ Division of Thoracic Surgery Keio University School of Medicine Shinjuku‐Ku Tokyo Japan; ^4^ Respiratory Medicine, National Hospital Organization, Kanagawa Hospital Hadano City Kanagawa Japan

**Keywords:** empyema, gentian violet, irrigation, methicillin‐resistant *Staphylococcus aureus*, pleural infection

## Abstract

Methicillin‐resistant *Staphylococcus aureus* (MRSA) empyema remains challenging despite guideline‐based management. Gentian violet (GV), an antiseptic dye, has been sporadically used for MRSA infections but not systematically evaluated in pleural disease. We analysed nine consecutive patients with MRSA empyema treated with intrapleural GV irrigation following surgical debridement between 2011 and 2018. All patients successfully completed GV irrigation without procedural complications. Bacterial clearance was achieved within a median of 5 days (IQR, 4–8), which was within the range observed in the non‐MRSA cohort treated with saline lavage (13 days [IQR, 6–24]). No GV‐related adverse events occurred during a median follow‐up of 993 days. Three patients developed *Pseudomonas aeruginosa* superinfection, likely reflecting GV's limited Gram‐negative activity. This case series demonstrates that intrapleural GV irrigation is feasible and safe, achieving microbiological clearance within an acceptable timeframe for MRSA empyema.

## Graphical abstract

This case series demonstrates that intrapleural gentian violet (GV) irrigation is a safe and effective adjunct for refractory MRSA empyema. GV irrigation achieved rapid bacterial clearance (median 5 days) without procedural complications or systemic toxicity, providing a viable alternative when standard surgical debridement and systemic antibiotics fail.

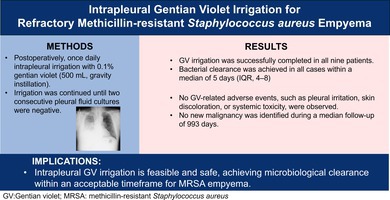

## 1 Introduction

Methicillin‐resistant *Staphylococcus aureus* (MRSA) remains a challenging pathogen in pleural empyema. Current international guidelines, including those from the Infectious Diseases Society of America, American Thoracic Society and British Thoracic Society, consistently recommend a combination of effective anti‐MRSA agents, such as vancomycin or linezolid and adequate pleural drainage [1, 2]. Despite these recommendations, some patients experience persistent infections refractory to conventional antibiotic therapy and drainage. These refractory cases are often associated with significantly prolonged hospitalisation and the potential need for invasive secondary interventions [3]. In such difficult‐to‐treat cases, the choice of additional interventions has largely relied on empiricism, with limited clinical evidence to guide practice.

Gentian violet (GV), an antiseptic dye with longstanding antimicrobial properties, has been used for MRSA infections in dermatology and otolaryngology, and sporadically as an adjunctive intrapleural therapy [4, 5]. However, its clinical efficacy and safety profile in pleural disease remain poorly defined, and GV is not addressed in major empyema management guidelines.

Our series analysed nine consecutive patients with refractory MRSA empyema treated with intrapleural GV irrigation following surgical debridement at a single institution between January 2011 and December 2018. The primary objective was to evaluate the feasibility and safety of intrapleural GV irrigation for MRSA empyema. Feasibility was defined by successful completion without procedural complications and achievement of bacterial clearance within a clinically acceptable timeframe. Safety was evaluated based on GV‐related adverse events. We compared outcomes descriptively with a reference cohort of 21 patients with non‐MRSA empyema treated with standard saline lavage.

## 2 Case Series

### 2.1 Patient Selection and Methods

This single‐center case series enrolled consecutive patients undergoing surgery for non‐fistulous empyema between January 2011 and December 2018 at National Hospital Organization Kanagawa Hospital (Kanagawa, Japan). Our series protocol was approved by the institutional review board (approval number: H300213). GV was used as an off‐label application after obtaining written informed consent from all patients regarding its use and potential risks. Refractory empyema was defined as non‐fistulous empyema with persistent fever, leukocytosis, or clinical deterioration despite appropriate antibiotic therapy and chest‐tube drainage.

The MRSA group (*n* = 9) comprised patients with non‐fistulous complex empyema, MRSA‐positive pleural fluid cultures who did not respond to medical therapy (antibiotic treatment plus chest‐tube drainage with persistent fever, leukocytosis, or clinical deterioration) and subsequently underwent pleural debridement or decortication, followed by postoperative intrapleural GV lavage. Among 110 consecutive patients surgically treated for non‐fistulous complex empyema, 21 patients with postoperative positive pleural cultures for non‐MRSA organisms (excluding fungus, tuberculosis and non‐tuberculous mycobacterial infection) who underwent postoperative saline lavage were used as a reference group [6]. Antibiotic regimens were individualised based on microbiological results and clinical response. Anti‐MRSA antibiotic therapy was continued until clinical improvement and microbiological clearance were achieved.

Empyema was defined according to international criteria as loculations or septations on computed tomography; gross pus or positive Gram stain/culture; or pleural fluid glucose < 60 mg/dL with LDH exceeding threefold the upper serum limit [7, 8]. Surgical indications included failure of antibiotic therapy and chest‐tube drainage or multi‐loculated. A 28‐Fr double‐lumen chest tube was inserted at surgery completion [6]. Postoperatively, patients in the MRSA group received once daily intrapleural irrigation with 0.1% gentian violet (500 mL, gravity instillation). Irrigation was continued until two consecutive pleural fluid cultures were negative. In cases with concomitant pneumonia, intravenous antibiotics were added, with vancomycin as the first‐line agent. In contrast, patients in the non‐MRSA group underwent twice‐daily intrapleural lavage with 1000 mL of warm saline. In both groups, daily pleural fluid cultures were obtained. Bacterial clearance was defined as two consecutive negative cultures. Chest tubes were removed after a 48‐h clamping trial if no clinical relapse occurred.

Variables included demographics, culture results, comorbidities, body mass index (BMI), prognostic nutritional index (PNI) and inflammatory markers (platelet‐to‐lymphocyte ratio; PLR). PNI was calculated as 10 × albumin (g/dL) + 0.005 × lymphocyte count (/μL) [9]. PLR was the absolute platelet count divided by the absolute lymphocyte count, calculated from blood samples taken at the time of empyema onset, which was an easily accessible marker of systemic inflammation. Charlson comorbidity index (CCI) was calculated without including age component.

### 2.2 Patient Characteristics

**FIGURE 1:**
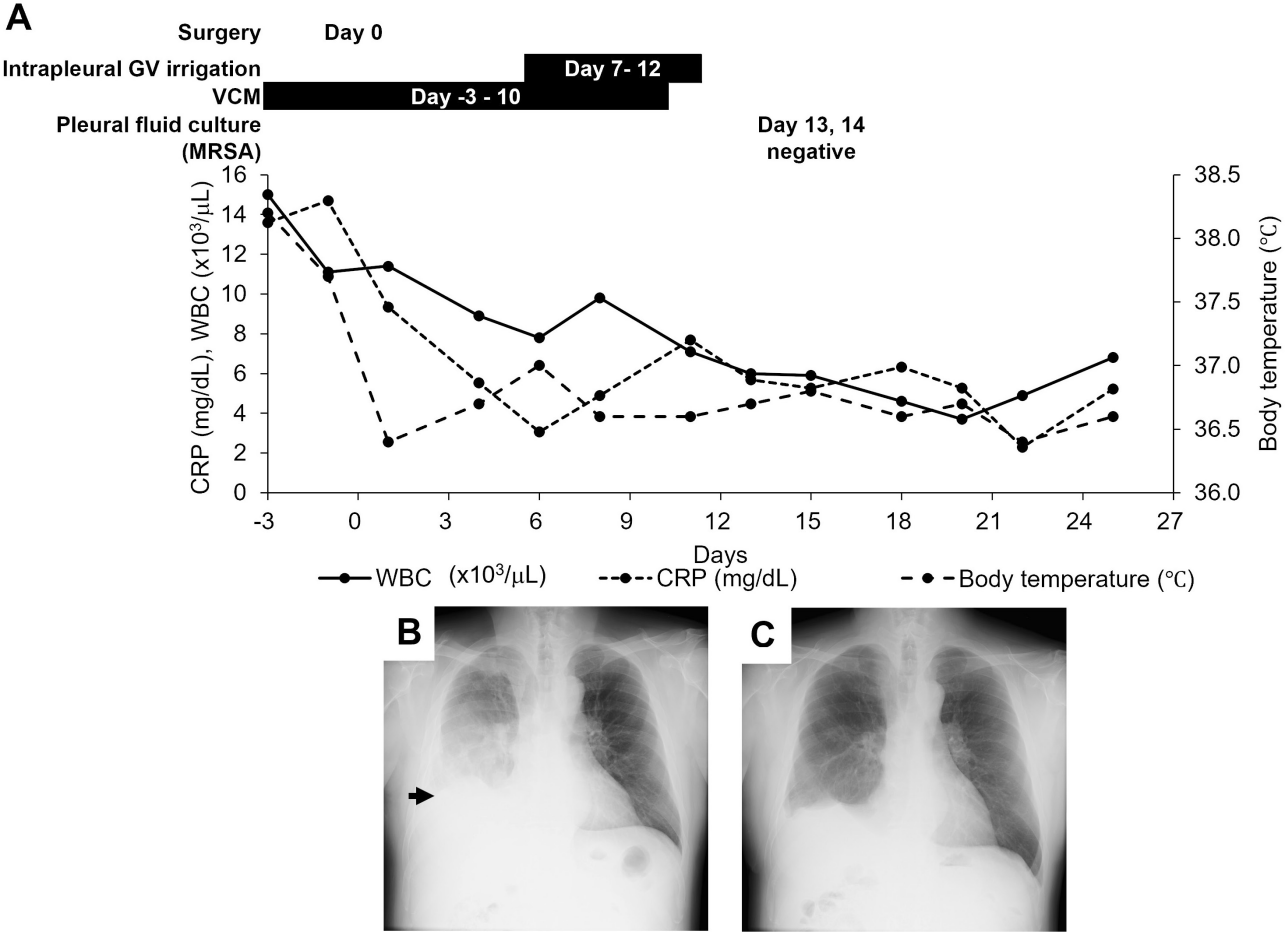
Clinical course and radiological findings of a representative case. (A) A 50‐year‐old male developed postoperative fever after video‐assisted thoracoscopic right upper lobectomy and lymph node dissection for lung cancer. Methicillin‐resistant *Staphylococcus aureus* (MRSA) was isolated from pleural fluid cultures, and pleural fluid analysis showed glucose 0 mg/dL and LDH 6180 U/L. Computed tomography revealed multiple‐loculated pleural effusion consistent with fibrinopurulent empyema. On day −3, a chest tube was inserted into the empyema cavity and antibiotic treatment was initiated. Surgical debridement was performed on Day 0. Postoperatively, after confirming that pleural fluid cultures remained positive for MRSA, intrapleural gentian violet irrigation was commenced and continued until two consecutive negative cultures were obtained. Subsequently, the patient's body temperature and laboratory findings improved. (B) Chest radiograph on Day 0 demonstrating right‐sided pleural effusion (arrow). (C) Chest radiograph eight months after treatment showing lung re‐expansion. CRP, C‐reactive protein; GV, gentian violet; MRSA, methicillin‐resistant *Staphylococcus aureus* ; VCM, vancomycin; WBC, white blood cell.

Thirty patients were enrolled (9 MRSA, 21 non‐MRSA). A representative case of MRSA empyema managed with intrapleural GV irrigation is shown in Figure 1. Median ages were 74 (IQR, 55–79) and 79 (IQR, 56–83), with male proportions of 100% and 57.1%, respectively. Most MRSA patients had performance status 0–1 (88.9% vs. 52.4%). Baseline parameters (MRSA vs. non‐MRSA): BMI, 23.7 (21.7–25.5) vs. 21.2 (19.5–23.7) kg/m^2^; albumin, 2.9 (2.7–3.3) vs. 2.1 (1.9–2.3) g/dL; leukocyte count, 8100 (4875–11,550) vs. 15,100 (7900–18,400)/μL; PNI, 37.3 (33.0–47.9) vs. 26.1 (23.1–28.7); neutrophil‐to‐lymphocyte ratio (NLR), 5.0 (1.9–7.1) vs. 11.2 (7.1–16.4); platelet‐to‐lymphocyte ratio (PLR), 201.4 (183.4–280.5) vs. 305.2 (263.7–410.4). CCIs were 2 (1–3) and 1 (1, 2), respectively. Albumin, PNI, NLR and PLR differed significantly between groups (Table 1).

**TABLE 1:** Baseline characteristics and clinical outcomes of patients with empyema.

Variables	MRSA empyema (*n* = 9)		Non‐MRSA empyema (*n* = 21)	
	Number or median	IQR or %	Number or median	IQR or %
Sex, male	9	100	12	57.1
Age, years	74	(55–79)	79	(56–83)
Smoking status				
Never smoker	6	66.7	5	23.8
Current or former smoker	3	33.3	2	9.5
Performance status				
0–1	8	88.9	11	52.4
2–4	1	11.1	10	47.6
Charlson comorbidity index	2	(1–3)	1	(1–2)
Clinical data				
BMI, kg/m^2^	23.7	(21.7–25.5)	21.2	(19.5–23.7)
Haemoglobin, g/dL	11.2	(10.8–11.6)	11.7	(10.7–12.7)
HbA1c, %	6.1	(6.0–7.0)	5.9	(5.8–6.3)
Albumin, g/dL	2.9	(2.7–3.3)	2.1	(1.9–2.3)
Leukocyte count, /μL	8100	(4875–11,550)	15,100	(7900–18,400)
PNI	37.3	(33.0–47.9)	26.1	(23.1–28.7)
NLR	5	(1.9–7.1)	11.2	(7.1–16.4)
PLR	201.4	(183.4–280.5)	305.2	(263.7–410.4)
Observation period, days	993	(547–1598)	87	(55–285)
Clinical outcomes				
Days to bacterial clearance from surgery	5	(4–8)	13	(6–24)
Days to chest tube removal	29	(19–64)	25	(18–35)
Length of hospital stay, days	82	(48–93)	45	(31–54)

Abbreviations: BMI, body mass index; MRSA, methicillin‐resistant *Staphylococcus aureus* ; NLR, neutrophil‐to‐lymphocyte ratio; PLR, platelet‐to‐lymphocyte ratio; PNI, prognostic nutritional index.

In the MRSA group, notable comorbidities included lung cancer (44%), prior pulmonary resection (33%), Parkinson's disease (22%) and diabetes mellitus (22%). In the non‐MRSA group, organisms included *Staphylococcus aureus* (28.6%), *Corynebacterium striatum* (28.6%), *Staphylococcus epidermidis* (19%) and Gram‐negative bacilli (19%).

Regarding the MRSA group, systemic vancomycin was used in four patients and linezolid in one. The median duration of these therapies was 8 days. In the remaining four patients, anti‐MRSA agents were not administered.

Intraoperative findings were comparable between groups, predominantly corresponding to fibrinopurulent (Stage II) empyema. The median operative duration in the MRSA group was 85 min (IQR, 43–177).

### 2.3 Outcomes

GV irrigation was successfully completed in all nine patients without procedural difficulties. No GV‐related adverse events, such as pleural irritation, skin discoloration, or systemic toxicity, were observed. No new malignancy was identified during a median follow‐up of 993 days. Bacterial clearance was achieved in all cases within a median of 5 days (IQR, 4–8), and chest tubes were removed at a median of 29 days (IQR, 19–64). For reference, in the non‐MRSA group treated with saline lavage, median time to bacterial clearance was 13 days (IQR, 6–24) and median time to chest‐tube removal was 25 days (IQR, 18–35). (Table 1).

Three MRSA patients developed *Pseudomonas aeruginosa* superinfection during GV irrigation, and one experienced delayed postoperative bleeding from an intercostal artery (day 32). In the non‐MRSA group, one patient required temporary mini‐tracheostomy for sputum retention.

Lung re‐expansion was achieved in all cases, as confirmed by follow‐up chest radiography.

## 3 Discussion

Our series provides clinical insights into the management of MRSA empyema by demonstrating the feasibility and safety of intrapleural GV irrigation. Current guidelines do not recommend intrapleural antibiotics or fibrinolytics as standard empyema therapy, and no specific recommendations exist for MRSA empyema [1, 2]. In this study, refractory empyema was defined as persistent pleural infection characterised by continued culture positivity and inflammatory response despite appropriate systemic anti‐MRSA therapy and surgical source control. Treatment remains challenging when systemic anti‐MRSA therapy and drainage fail. Previous studies have reported that reintervention after surgery occurs in approximately 10% of cases, particularly in infections caused by resistant organisms such as MRSA [10]. This condition is associated with prolonged hospitalisation, extended courses of antibiotics, and the need for invasive procedures like open‐window thoracostomy, all of which adversely affect patient outcomes [3]. As illustrated in our representative case (Figure 1), even when intraoperative findings show fibrinopurulent changes (Stage II), MRSA can be difficult to eradicate through mechanical debridement alone. In such contexts, prompt bacterial clearance (median 5 days) without GV‐related toxicity suggests that GV irrigation is a viable adjunctive option.

GV, also known as hexamethyl pararosaniline, exerts broad antimicrobial effects, particularly against Gram‐positive bacteria like MRSA by disrupting redox homeostasis via covalent modification of thioredoxin reductase [11, 12]. Although topical GV use has been validated in other clinical fields, its systematic evaluation in thoracic infections was previously lacking [4].

Intrapleural GV irrigation achieved bacterial clearance within a median of 5 days without procedural complications. This timeframe and the subsequent clinical course, including chest‐tube duration and hospitalisation, were within a similar range to those observed in the non‐MRSA group treated with standard saline lavage.

A strength of our series lies in its assessment of feasibility and safety. No patient developed pleural irritation, skin toxicity or systemic complications, and no malignancies were observed during a median follow‐up of 3 years, despite its IARC Group 2B classification [13]. The selection of patients was strictly limited to those failing standard management, ensuring an acceptable risk–benefit profile. While secondary *Pseudomonas aeruginosa* colonisation occurred in three cases, it reflects GV's limited activity against Gram‐negative bacteria rather than a direct adverse effect.

Our series has limitations, including its small, single‐centre, non‐comparative design. Larger controlled studies are needed to establish standardised treatment protocols.

In conclusion, intrapleural GV irrigation following surgical debridement was feasible and safe, achieving timely microbiological clearance in MRSA empyema. GV may serve as a supervised adjunctive option when standard therapy fails.

## Author Contributions

All authors meet the ICMJE authorship criteria and approve the final version of the manuscript.

## Funding

The authors have nothing to report.

## Ethics Statement

Our series protocol was approved by the institutional review board (approval number: H300213). GV was used as an off‐label application after obtaining written informed consent from all patients regarding its use and potential risks.

## Consent

The authors declare that written informed consent was obtained for the publication of this manuscript and accompanying images and attest that the form used to obtain consent from the patients complies with the journal requirements as outlined in the author guidelines.

## Conflicts of Interest

The authors declare no conflicts of interest.

## Data Availability

The data that support the findings of this study are available from the corresponding author upon reasonable request.
